# DFT Investigation of Substitutional and Interstitial Nitrogen-Doping Effects on a ZnO(100)–TiO_2_(101) Heterojunction

**DOI:** 10.1021/acs.jpcc.1c09395

**Published:** 2022-02-03

**Authors:** Ida Ritacco, Olga Sacco, Lucia Caporaso, Matteo Farnesi Camellone

**Affiliations:** † Dipartimento di Chimica e Biologia, Università degli Studi di Salerno, via Giovanni Paolo II 132, 84084 Fisciano, Salerno, Italy; ‡ CNR-IOM, Consiglio Nazionale delle Ricerche - Istituto Officina dei Materiali, c/o SISSA, 34136 Trieste, Italy

## Abstract

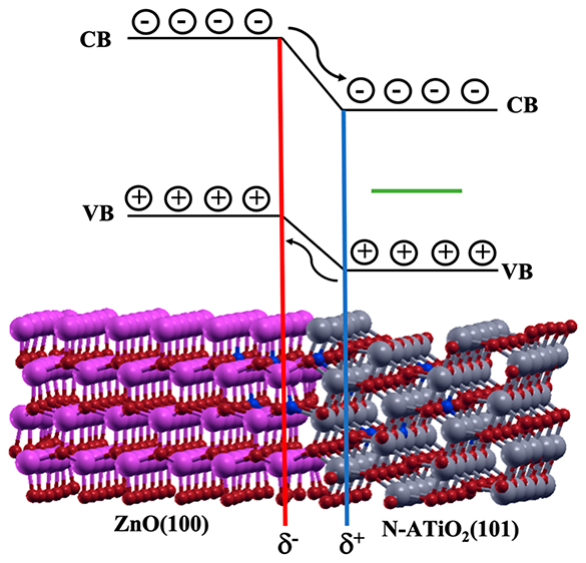

Density Functional Theory (DFT) calculations have been performed to investigate the structural and electronic properties of the ZnO(wurtzite)–ATiO_2_(anatase) heterojunction in the absence and presence of substitutional, interstitial nitrogen (N) doping and oxygen vacancies (O_V_). We report a detailed study of the interactions between the two nonpolar ZnO and TiO_2_ surfaces and on the role of N-doping and oxygen vacancies, which are decisive for improving the photocatalytic activity of the heterojunction. Our calculations show that substitutional N-doping is favored in the ATiO_2_ portion, whereas the interstitial one is favored in the ZnO region of the interface. Both substitutional and interstitial N-doped sites (i) induce gap states that act as deep electronic traps improving the charge separation and delaying electron–hole recombination, (ii) facilitate the O_V_ formation causing a decrease in the formation energy (*E*_FORM_), and (iii) do not affect the band alignment when compared to the undoped analogue system. The presented results shed light on the N-doping effect on the electronic structure of the ZnO(100)–TiO_2_(101) heterojunction and how N-doping improves its photocatalytic properties.

## Introduction

Titania (TiO_2_) is a material widely employed in material science applications, such as environmental cleanup,^1^ catalysis, and photocatalysis, due to its excellent photoactivity, stability over time, low costs, and no toxicity.^2,3^ Being a semiconductor with a large band gap (*E*_g_ = 3.23 eV), TiO_2_ exhibits photocatalytic activity under UV light irradiation (λ < 384 nm), which represents a small portion of solar energy (∼5%), while it is not very effective under visible light, which instead represents an important part of solar energy (∼45%).^4^

Recent studies have suggested different approaches to improve (i) the chemical–physical properties of TiO_2_ and (ii) its performances under visible light, such as doping with nitrogen (N) atoms^5−8^ and the formation of a heterojunction with other oxides,^9−11^ such as zinc oxide (ZnO).^12−14^ In fact, the presence of N dopants improves the TiO_2_ absorption in the visible light^15,16^ by forming band gap states that trap the photogenerated electrons, while the heterojunctions delay the rapid recombination holes–electrons. Therefore, the concerted use of heterojunctions and N-doping is, to date, the winning strategy to improve the photoactivity of the semiconductor systems.^17−20^ Previous experimental studies^21,22^ have revealed that ZnO–TiO_2_ heterojunctions reduce the electronic recombination since the TiO_2_ and ZnO interfaces trap the photogenerated electrons and holes that are transferred to adsorbed species, initiating surface reduction/oxidation reactions.^23−25^ Therefore, the study of the structural and chemical–physical properties of the undoped and N-doped ZnO–TiO_2_ heterojunction is fundamental to obtaining a semiconductor with improved photocatalytic activity.

Here, we investigate the structural and electronic properties of the heterojunction between wurtzite (ZnO) and anatase (ATiO_2_) both in the presence and absence of N dopants by using density functional theory (DFT). For all systems, we have determined the valence band and conduction band offsets (VBO and CBO, respectively) as well as the dipole that forms at the interface in order to predict the charge carriers migration and the type of the heterojunction. In the doped heterojunctions, the energetically preferred location of substitutional and interstitial N dopants^5,6,26^ and the role played by the N concentration on the band alignment have been investigated. The band offsets are fundamental parameters to determine the electronic transport and the behavior of metal oxides during the formation of the heterojunction.^27−29^ In addition, we have computed the formation energies (*E*_FORM_) of oxygen vacancies (O_V_) both in the undoped and N-doped systems. It is found, in agreement with similar studies, that the presence of the N dopant stabilizes the formation of O_V_.^20^ Structural and electronic analyses indicate that the improved catalytic performance of the heterojunction with respect to the two separate materials depends on (i) the interface dipole, generated in the contact area of the heterojunction and in (ii) the presence of N-doped sites, most favored in the anatase region. In fact, while the dipole nature induces a flow of the photogenerated electrons from ZnO to TiO_2_, the N-doped sites generate empty states in the anatase capable of trapping these electrons, thus, delaying electron–hole recombination.

## Computational Details

Density functional theory (DFT) calculations have been performed using the Quantum-ESPRESSO computer package.^30^ The exchange and correlation energy functional expressed in the Perdew–Burke–Ernzerhof (PBE) generalized gradient approximation (GGA)^31^ has been employed. The spin-polarized Kohn–Sham equations were solved in the plane-wave pseudopotential framework, with the wave function basis set and the Fourier representation of the charge density being limited by kinetic cutoffs of 40 and 250 Ry, respectively. The Ti, Zn, O, and N atoms were described by ultrasoft pseudopotentials.^32^ The valence electron configurations of Ti, Zn, O, and N are [Ar] 3d^2^4s^1^, [Ar] 4s^2^4p^0.3^3d^9.7^, [He] 2s^2^4p^4^, and [He] 2s^2^2p^3^, respectively. It has been shown that the addition of a Hubbard U^33^ term acting on the Ti 3d orbitals allows a more accurate description of the electronic structure of TiO_2_^34^ not affecting the computational cost compared to a conventional DFT-GGA functional. We used a value of *U* = 3.9 eV on TiO_2_ portion.^1,34,35^ The use of the DFT+*U* method on the ZnO region generates problems in the convergence system due to the large size of the heterojunction. Therefore, we preferred not to apply a Hubbard *U* term on Zn, in line with other previous theoretical work.^36−40^

In order to design a realistic model system of the ZnO–TiO_2_ heterojunction, we have considered the wurtzite (100) and the anatase (101) nonpolar surfaces, being the most stable terminations as suggested from experiments (XRD analysis) and theory.^36,41−43^

The computed lattice parameters for hexagonal wurtzite bulk and tetragonal anatase bulk are *a*_ZnO_(*X*) = 3.25 Å, *b*_ZnO_(*Y*) = 5.21 Å and *a*_ATiO2_(*X*) = 3.78 Å, *b*_ATiO2_(*Y*) = 9.49 Å, respectively. These lattice parameters are in very good agreement with the experimental ones, *a*_ZnO_(*X*) = 3.2496 Å, *b*_ZnO_(*Y*) = 5.2042 Å^44^ and *a*_ATiO2_(*X*) = 3.7848 Å, *b*_ATiO2_(*Y*) = 9.5123 Å.^45^

**Figure 1:**
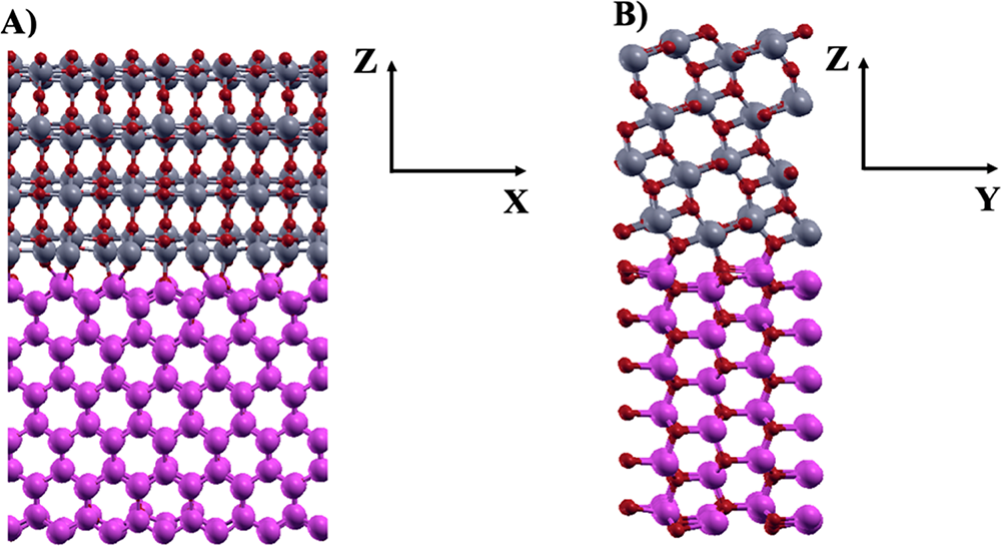
Optimized structure of the undoped ZnO(100)–ATiO_2_(101) interface, orientation along (A) *XZ* and (B) *YZ*. Zn, O, and Ti atoms, purple, red, and gray, respectively, are represented in ball and sticks.

In line with previous studies,^46^ we have built our interface model system by matching a (6 × 2) supercell of ZnO (100) with a (5 × 1) supercell of ATiO_2_ (101) containing, in the *Z* direction, six bilayers and four layers, respectively (see Figure 1). Previous studies have shown that this number of layers is sufficient to simulate accurate and converged surface properties.^46−49^ Since the supercell sizes are equal to *X*_ATiO2_ = 5*a*_ATiO2_, *X*_ZnO_ = 6*a*_ZnO_, *Y*_ATiO2_ = *b*_ATiO2_ and *Y*_ZnO_ = 2*b*_ZnO_, the lattice mismatch deriving from the combination of the two surfaces is around 3% along the *X* direction and 9% along the *Y* direction: (Δ*X*/*X*_ATiO2_) ≈ (Δ*X*/*X*_ZnO_) ∼ ±3.2–3.1% and (Δ*Y*/*Y*_ATiO2_) ≈ (Δ*Y*/*Y*_ZnO_) ∼ ±9.8–8.9%.

Our model consists of a total number of 528 atoms of which 288 of ZnO ((ZnO)_144_ units) and 240 of ATiO_2_ ((TiO_2_)_80_ units). Due to the large size of the system, the Brillouin zone integration has been performed on the Γ point only.

Several tests were performed to identify the most stable configurations of the heterojunction. These tests were carried out by displacing the positions of ATiO_2_ atoms by 0.33 Å along the *X* direction until they coincide with the equivalent positions and fixing the positions of ZnO atoms. Subsequently, substitutional (interstitial) N-doping was simulated by replacing (adding) one, two, or three oxygen atoms in the ZnO and ATiO_2_ side of the heterojunction with nitrogen atoms.

The valence and conduction band offsets (VBO and CBO, respectively) of the undoped and N-doped ZnO(100)–ATiO_2_(101) interfaces have been computed following the method described in refs (36, 50, and 51) and using the following equations:
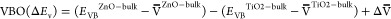
1
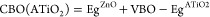
2In eq 1, *E*_VB_ and V̿ correspond to the valence band energy value, defined by Projected Density of State (PDOS) analyses, and the macroscopically averaged potentials obtained from two independent ZnO and TiO_2_ bulk calculations, while the term ΔV̿ results from the lineup of the macroscopic average of the electrostatic potential across the heterojunction. In eq 2, we use the experimental band gap (Eg) of ZnO (3.4 eV) and ATiO_2_ (3.2 eV) due to the limitations of DFT calculations in quantitatively defining band gaps.

Defining the band offsets, it is possible to obtain the “Minimal Band Gap” (MBG), corresponding to the interface gap, through the following equation:
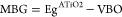
3The interface dipole in the undoped heterojunction has been evaluated by means of the plot of the charge density difference (bonding charge analysis) and the plane-averaged charge density difference along the *Z* direction (Δρ_*z*_), showing the nature and the direction of the interface polarization.

The energy gain deriving from the formation of the undoped heterojunction from the separated ZnO and ATiO_2_ slabs and defined as the adhesion energy *E*_ad_ has been computed through the formula:
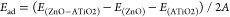
4where *A* is the surface area, *E*_(ZnO-ATiO2)_ is the energies of the undoped ZnO(100)–ATiO_2_(101) heterojunction and *E*_(ZnO)_ – *E*_(ATiO2)_ are the energies of the isolated slabs ZnO and ATiO_2_, respectively.

The formation energies of oxygen vacancies have been determined according to the following equation:
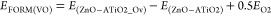
5*E*_(ZnO-ATiO2_Ov)_ is the energy of the undoped (N-doped) ZnO(100)–ATiO_2_(101) heterojunction when oxygen vacancies are present, while *E*_(ZnO-ATiO2)_ and *E*_O2_ are the energies of the nondefective undoped (N-doped) ZnO(100)–ATiO_2_(101) heterojunction and the gas phase O_2_ molecule, respectively.

## Results and Discussion

### Undoped ZnO(100)–ATiO_2_(101) Heterojunction

To create the interface between the TiO_2_ and ZnO oxides, we have selected the most representative (101) anatase (ATiO_2_) termination and the (100) wurtzite (ZnO) termination, as suggested by previous experimental and theoretical studies.^20,36,41−43,46,52^ For this purpose, a (5 × 1) supercell of ATiO_2_ (101) consisting of four layers is put in contact with a (6 × 2) supercell of ZnO(100) consisting of six bilayers. Since the anatase supercell is larger in size than that of wurtzite, the lattice parameters of anatase ATiO_2_(101) have been rescaled with respect to those of ZnO(100) along the interfacial plane. The optimized structure of the ZnO(100)–ATiO_2_(101) interface, depicted in Figure 1, is composed of 528 atoms, 240 belonging to the ATiO_2_(101) surface and 288 to the ZnO(100) surface. The lattice parameters of the supercell are 19.7 Å, 10.6 Å, and 32.0 Å along *a*(*X*), *b*(*Y*), and *c*(*Z*) directions, respectively.

**Figure 2:**
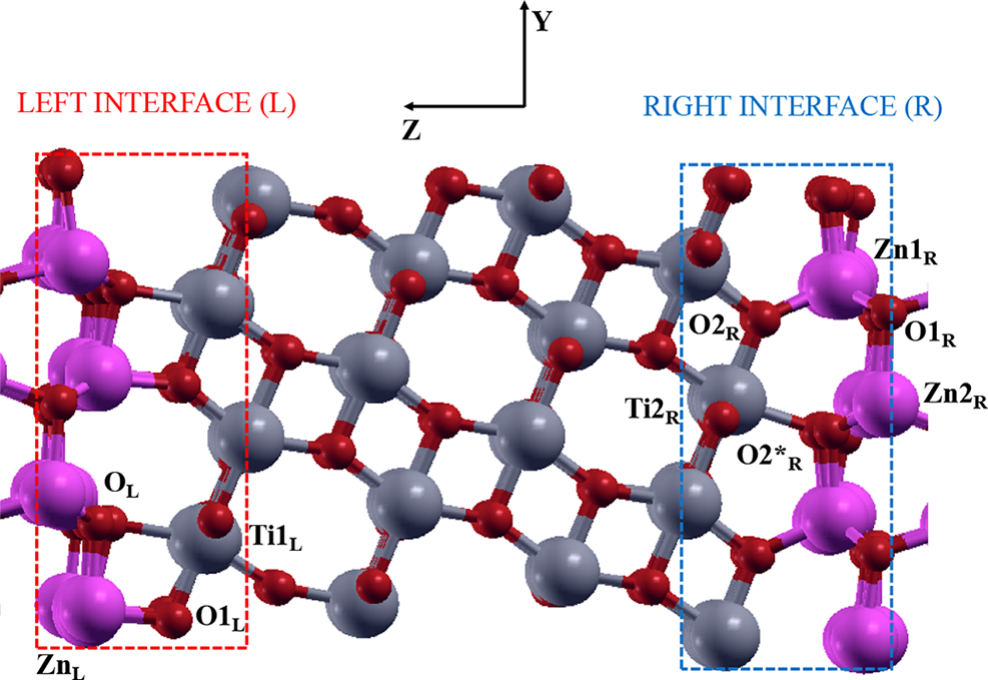
Different relaxations of the left (L, red dashed square) and right (R, blu dashed square) interface. Zn, O, and Ti atoms, purple, red, and gray, respectively, are represented in ball and sticks.

When periodic boundary conditions are applied along the *Z* direction of our model system, it is possible to identify two different ZnO(100)–ATiO_2_(101) interfaces, one on the left (L) and the other one on the right (R) side of the supercell (see Figure 2).

Upon relaxation, the oxygen atoms, O_L_ of the left interface, belonging to the ZnO(100) surface, relax inward and coordinate the Ti1_L_ atoms of the nearby ATiO_2_(101) surface, whereas the O1_L_ atoms of the ATiO_2_(101) surface bind the Zn_L_ atoms of the ZnO(100) surface. As a result, the left ZnO(100)–ATiO_2_(101) interface exhibits a rhombohedron-like structure involving Zn_L_-O_L_-Ti1_L_-O1_L_ atoms. At the right interface, the O2*_R_ atoms of the wurtzite surface relax outward, binding the Ti2_R_ atoms of the titania, while the O2_R_ atoms of the ATiO_2_(101) surface coordinate the Zn1_R_ atoms of the wurtzite, which are pushed toward the ZnO surface. From this interaction, on the right heterojunction, a nonregular hexagonal-like lattices is generated between Ti2_R_-O2_R_-Zn1_R_-O1_R_-Zn1_R_-O2*_R_ atoms.

The computed adhesion energies (*E*_ad_) of the right (−0.39 Jm^–2^) and left (−0.36 Jm^–2^) interface are almost the same,^29,53^ while the total energies of the two separate interfaces suggest that the right is more stable than the left one by 0.8 eV. Therefore, in the following we focus on the electronic properties and on the energy band alignment of the most stable ZnO(100)–ATiO_2_(101) right interface.

At the metal/oxide interface, the energy bands of ZnO and ATiO_2_ come together, and since energy bands of the two materials are positioned discontinuously from each other, they align at the interface. In fact, the band offsets represent the alignment of energy bands at the heterojunction.

The valence (VBO) and conduction (CBO) band offsets of the ZnO(100)–ATiO_2_(101) heterojunction have been computed using eqs 1 and 2, respectively.

**Figure 3:**
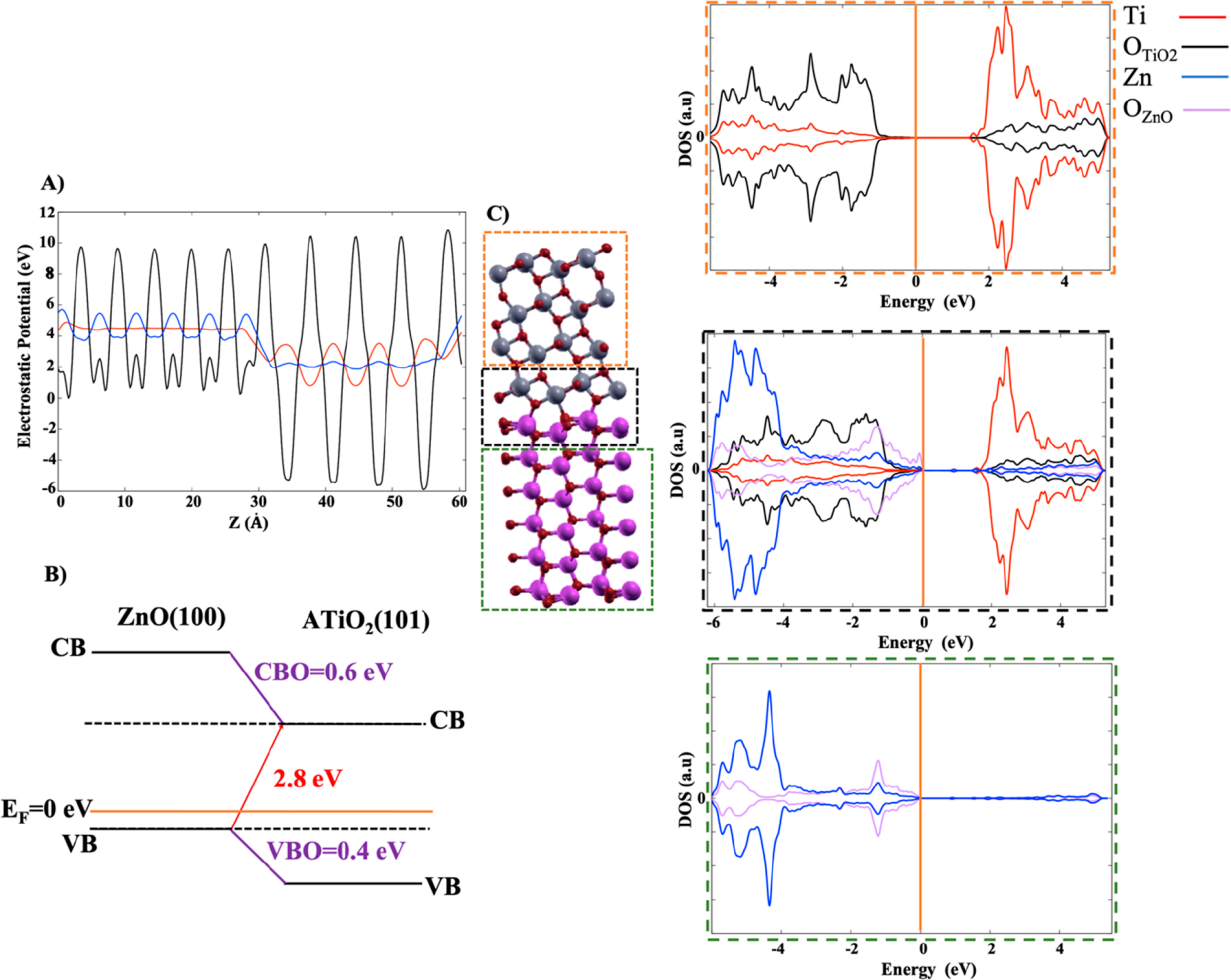
(A) Planar-averaged electrostatic potential (black line) and its macroscopic average for the ZnO(100)-ATiO_2_(101) heterojunction (red and blue lines, respectively). (B) Band alignment of ZnO(100)–ATiO_2_(101) heterojunction and (C) graphical representation of ATiO_2_, ZnO(100)–ATiO_2_(101) interface, and ZnO regions outlined in orange, black, and green dashed squares, respectively, along with the PDOS. The states of titanium (Ti) and oxygen (O) in ATiO_2_ and ZnO(100)–ATiO_2_(101) interface regions are represented in red and black, respectively, while the states of zinc (Zn) and oxygen (O) in ZnO and ZnO(100)–ATiO_2_(101) interface regions are represented in blue and purple. On the DOS (au) axis, the values above and below 0 au indicate the electrons with spin up and spin down, respectively. The Fermi energy level is represented with an orange line at 0 eV.

To determine the VBO and CBO of the interface, the energy values of the valence band (E_VB_), of the macroscopically averaged potentials V̿ of ZnO and TiO_2_ bulks as well as of the term ΔV̿ resulting from the lineup of the macroscopic average of the electrostatic potential across the heterojunction have been computed (see Table 1 and Supporting Information (SI)). Figure 3A shows a plot of the electrostatic (black line) and the macroscopically averaged (blue and red lines) potentials of the ZnO(100)–ATiO_2_(101) heterojunction along the direction perpendicular (*Z*) to the interface. The lineup of the macroscopically averaged electrostatic potential across the interface gives ΔV̿ = 2.4 eV.

**Table 1:** Energy Values of the Valence Band (*E*_VB_) and of the Macroscopically Averaged Potentials (V̿) of ZnO and TiO_2_ Bulks, as Well as the Term ΔV̿ Resulting from the Lineup of the Macroscopic Average of the Electrostatic Potential across the Heterojunction

	ZnO	TiO_2_	ZnO(100)–ATiO_2_(101)
*E*_VB_ (eV)	7.3	7.7	
V̿ (eV)	4.5	2.9	
ΔV̿ (eV)			2.4

Replacing the values reported in Table 1 into eq 1, we obtain a VBO of 0.4 eV, which is in good agreement with the experimental value of 0.53 ± 0.07 eV.^54,55^ Finally, using eq 2, a CBO of 0.6 eV is predicted. These values indicate that the anatase band edges are lower in energy than those of wurtzite. Therefore, the band alignment of the undoped interface is typical of a semiconductor heterojunction type II (see Figure 3B), in agreement with the experimental evidence,^22,54,56−59^ in which the photogenerated electrons should migrate from CB of ZnO to CB of ATiO_2_, while the holes from VB of ATiO_2_ migrate to VB of ZnO. The interface minimal band gap, computed using eq 3, is 2.8 eV (Figure 3B).

Figure 3C shows the PDOS of the undoped ZnO(100)-ATiO_2_(101) interface. In order to understand the contribution of the different atoms of the interface to the PDOS, we have divided the supercell into three regions: (i) the interfacial region consisting of one ATiO_2_(101) trilayer and one ZnO(100) bilayer (black dashed square of Figure 3C), (ii) the ATiO_2_-like region (orange dashed square of Figure 3C), (iii) and the ZnO-like region (green dashed square of Figure 3C).

The PDOS of the three different regions of the heterojunction do not exhibit peaks within the band gap. Furthermore, a closer inspection to the PDOS shows that at the interfacial region the valence (VB) and conduction (CB) bands of ZnO are higher in energy than the band edges of ATiO_2_, confirming qualitatively and not quantitatively, due to hybridization phenomena that could influence the VB and CB positions, the band alignment obtained for the undoped heterojunction and the flow of electrons (holes) from wurtzite (anatase) to anatase (wurtzite) and vice versa (compare Figure 3B and C).

**Figure 4:**
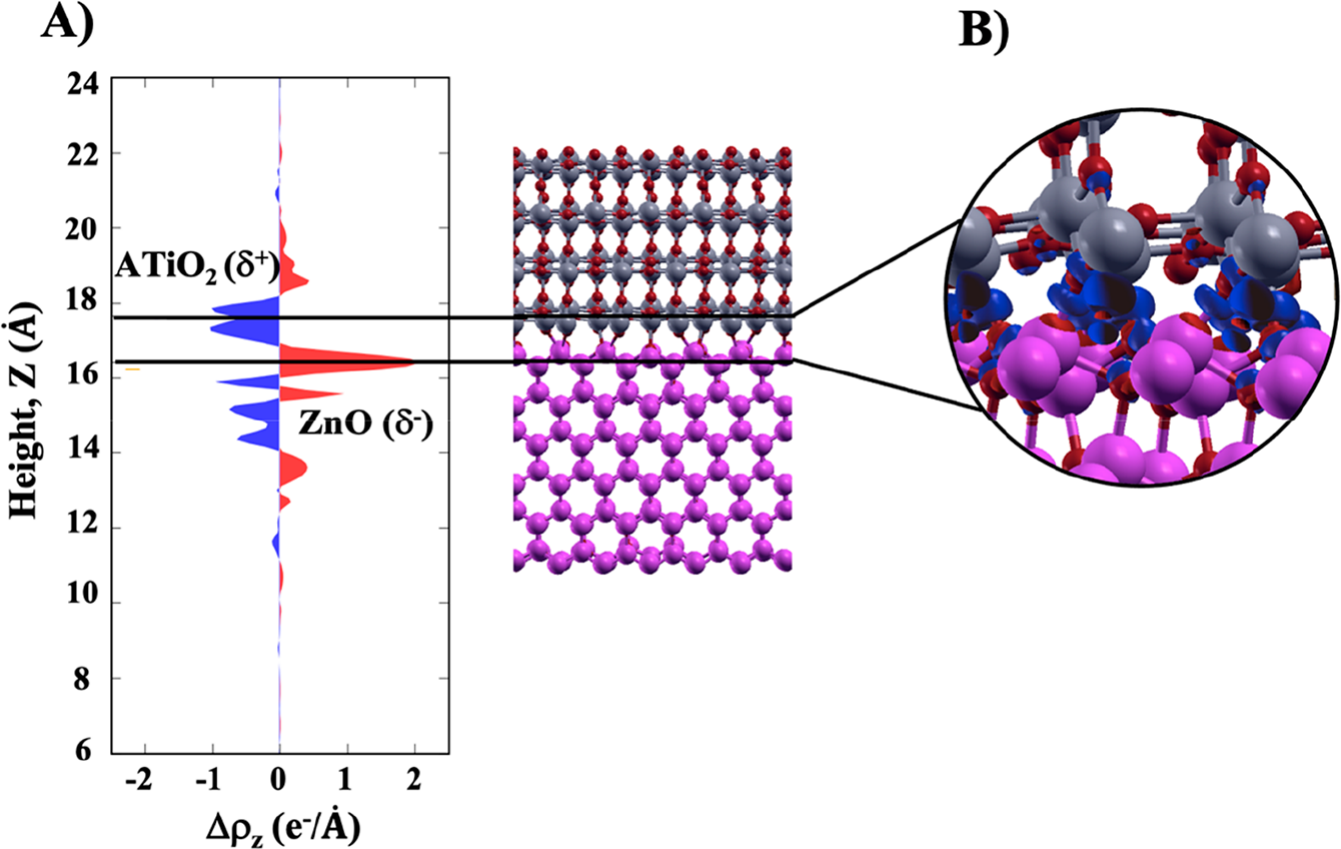
(A) Plane-averaged charge density difference along the *Z* direction (Δρ_*z*_) and (B) bonding charge analysis. In both analyses, the charge accumulation on ZnO (δ^–^) and the depletion charge on ATiO_2_ (δ^+^) are indicated in red and blue, respectively.

The electrons migration from ZnO to ATiO_2_ was further confirmed by the plot of the charge density difference (bonding charge analysis) and the plane-averaged charge density difference along the *Z* direction (Δρ_*z*_; see Figure 4A and B, respectively).^27−29^ These analyses show the formation of a dipole at the ZnO(100)–ATiO_2_(101) interface directed from the wurtzite to the anatase, indicating a charge accumulation (δ^–^) on ZnO and a charge depletion (δ^+^) on ATiO_2_.

Therefore, the interface dipole nature confirms the formation of a type II heterojunction in which the photoexcited electrons flow from wurtzite to anatase.

### Substitutional and Interstitial N-Doping of the ZnO(100)–ATiO_2_(101) Heterojunction

Moving on the effects of nitrogen (N)-doping on the structural and electronic properties of the ZnO(100)–ATiO_2_(101) heterojunction, we have considered the doping of the system with substitutional as well as with interstitial N.

To study the substitutional N-doping, we have considered the optimized structure of the ZnO(100)–ATiO_2_(101) heterojunction and replaced one oxygen (O) atom of the ZnO and the ATiO_2_ region of the interface with a N atom.

Considering only the oxygen atoms of wurtzite and anatase, 144 for ZnO(100) and 160 for ATiO_2_(101), the addition of a single N atom in the ZnO region correspond to 0.69% of nominal doping ratio, while in ATiO_2_ region correspond to 0.63% of nominal doping ratio, in agreement with the experimental range (0.5–2%) estimated by XPS analyses in ref (60). Furthermore, being N an atom with five valence electrons ([He] 2s^2^2p^3^), its presence in the supercell carries one unpaired electron.^5^

**Figure 5:**
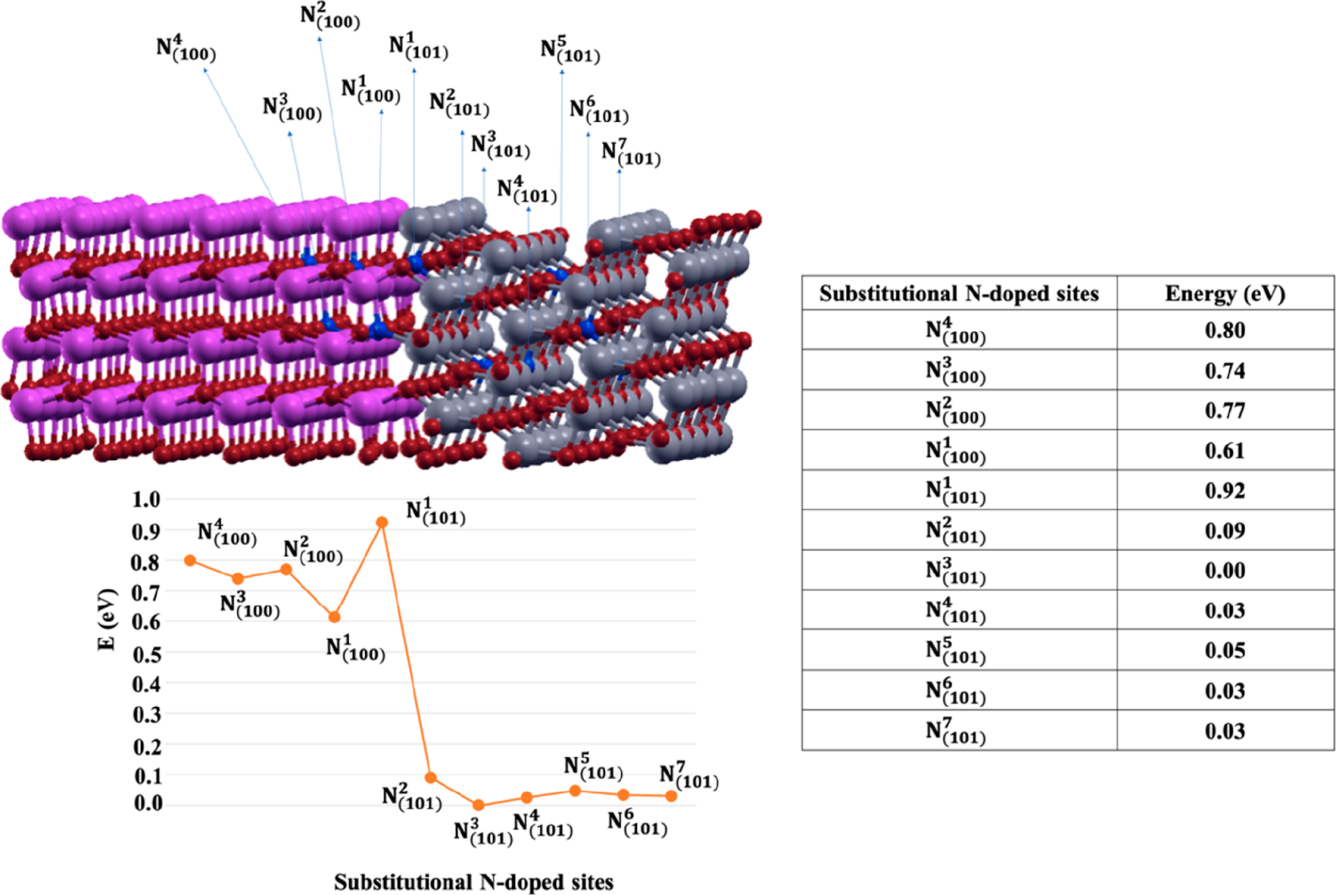
Substitutional N-doped sites in ATiO_2_(101) and ZnO(100) regions of the heterojunction with the corresponding relative energies in eV. The zero in energy is N_(101)_^3^ that corresponds to the most stable N-doped site.

In our calculations, we have considered seven and four N-doped sites for ATiO_2_(101) and ZnO(100), respectively. The position of the different N-doped sites in the ZnO(100)–ATiO_2_(101) heterojunction and the corresponding energies computed with respect to the most stable N-doped system are reported in Figure 5.

**Figure 6:**
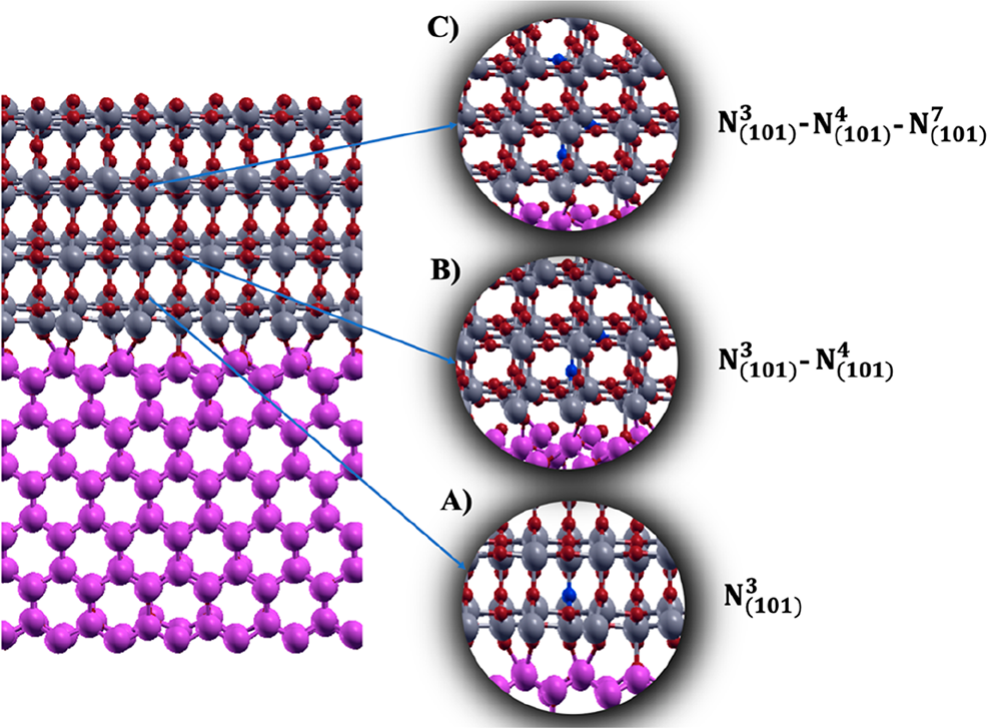
N-doped ZnO(100)–ATiO_2_(101) heterojunction with (A) one, (B) two, and (C) three substitutional N-doped sites.

From the Table in Figure 5 it clearly emerges that the doping with N in the ATiO_2_ (101) portion of the heterojunction (N_(101)_^1, ..., 7^)) results in structures whose energies decrease moving from the contact region toward the inside of the ATiO_2_ region (from 0.9 to 0.03 eV). The lowest energy value was obtained for N_(101)_^3^, where a N atom replaced an O atom of the interfacial ATiO_2_(101) bilayer further away (Figure 6A). Instead, N-doping of the ZnO (100) side gives structures (N_(100)_^1, ..., 4^)) always higher in energy (from 0.6 to 0.8 eV) than those obtained for (N_(101)_^2, ..., 7^)) models.

N_(101)_^1^ and (N_(100)_^1, ..., 4^)) are energetically unstable due to structural rearrangements induced by the substitutional N, that is, the breaking of Zn–O bonds near to the dopants, which do not occur moving away from the interfacial zone.

In order to investigate the effect of the dopant concentration on the band offsets calculations and on the electronic structure of the N-doped heterojunction, we considered one, two and three N-doped sites in the ATiO_2_ supercell, corresponding to 0.63%, 1.26% and 1.89% doping ratio, respectively. In particular, together with N_(101)_^3^, we have considered also the energetically stable N_(101)_^4^ and N_(101)_^7^ sites obtaining two additional configurations defined for clarity N_(101)_^3^–N_(101)_^4^ and N_(101)_^3^–N_(101)_^4^–N_(101)_^7^ (Figure 6B and C, respectively).

We now investigate the interstitial N-doping adding a N atom in the ZnO and ATiO_2_ part of the optimized undoped heterojunction.

Previous experimental and theoretical studies have shown that the doping of ATiO_2_ with interstitial N leads to the formation of N–O species with N bonded to a lattice oxygen atom.^5,26^ Therefore, in agreement with results reported in literature,^5,26^ we have identified three different sites for the interstitial N-doping in the ATiO_2_(101) portion.

**Figure 7:**
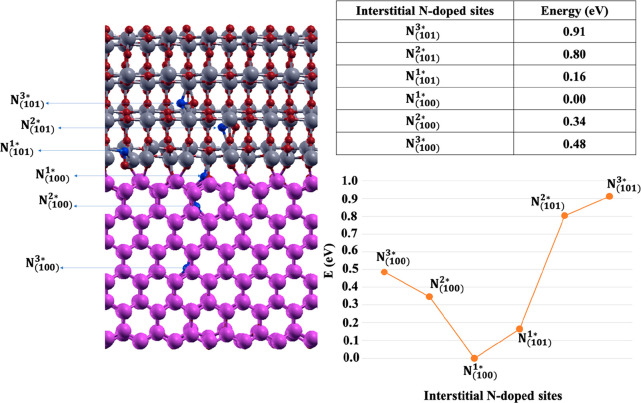
Interstitial N-doped sites in ATiO_2_(101) and ZnO(100) regions of the heterojunction with the corresponding relative energies in eV. The zero in energy is N_(100)_^1_*_^ that corresponds to the most stable N-doped site.

Similarly to ATiO_2_(101), also for the ZnO(100) we have identified three interstitial N-doping sites moving from the contact region of the two surface to the inside of the ZnO portion.^61^ The corresponding configurations and relative energies are shown in Figure 7.

Considering all atoms of wurtzite and anatase, 288 for ZnO(100) and 240 for ATiO_2_(101), the addition of a single N interstitial atom in the ZnO region correspond to 0.35% of nominal doping ratio, while in ATiO_2_ region correspond to 0.41% of nominal doping ratio.

In contrast to the substitutional N-doping case, the interstitial N-doped sites of ZnO(100) are more stable than that of ATiO_2_(101). Indeed, in ZnO(100) interstitial N generates structural rearrangements resulting in a twisted bond Zn–N–O–Zn in which Zn maintain their stable tetrahedral structure.^61^ While the Ti atoms of TiO_2_ (101) portion after the coordination with the interstitial N lose their stable octahedral geometry due to an increase of the coordination number.

In addition, the energy of the doped interface increases when the interstitial N atom is placed farther from the interfacial region (see Table in Figure 7).

Despite the lowest energy configuration is N_(100)_^1_*_^, belonging to the interfacial bilayer ZnO(100), we considered only the interstitial N-doping of the ATiO_2_ portion. This choice originate from previous literature studies, showing how the N-doping of ZnO prevents the formation of high hole concentrations at room temperature inhibiting the photocatalytic activity of the system.^62,63^

**Figure 8:**
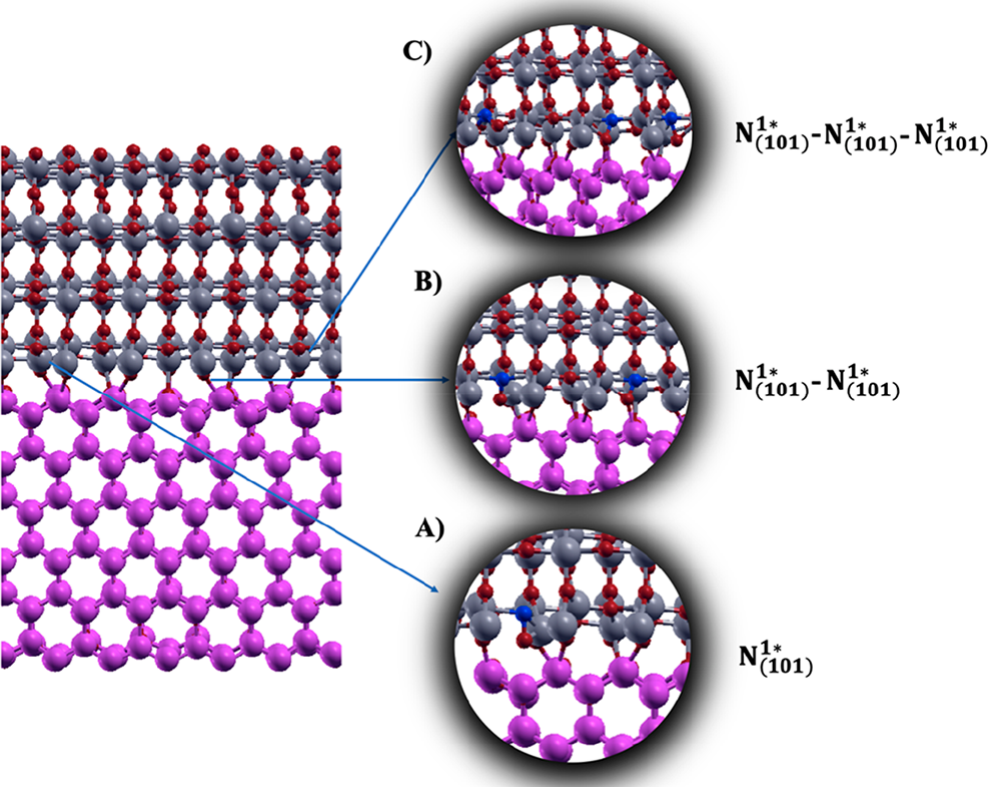
N-doped ZnO(100)–ATiO_2_(101) heterojunction with (A) one, (B) two, and (C) three interstitial N-doped sites.

Starting from the most stable configuration of ATiO_2_(101) labeled N_(101)_^1*^ (see Figure 8A), the effect of the N dopant concentration on the heterojunction properties has been investigated considering two additional configurations with two and three interstitial N atoms in the ATiO_2_ region, defined for clarity N_(101)_^1_*_^–N_(101)_^1_*_^ and N_(101)_^1_*_^–N_(101)_^1_*_^–N_(101)_^1_*_^ (see Figure 8B,C). In the three interstitial N-doped heterojunctions, the nitrogen percentage corresponds to 0.41%, 0.82%, and 1.23% doping ratio, respectively.

In all optimized model systems, the N–O distance is always within 1.32 and 1.35 Å (depending on the model considered), indicating the presence of a N–O bond, again in agreement with the literature’s data for N-doped ATiO_2_ (1.36 Å).^6^

### Band Offsets and Electronic Properties of Substitutional and Interstitial N-Doped ZnO(100)–ATiO_2_(101) Interface

**Figure 9:**
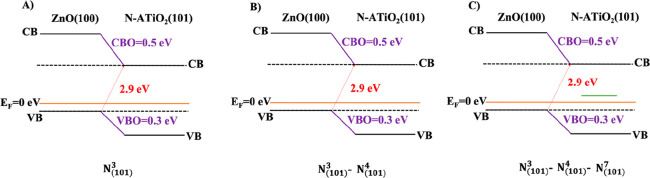
Band alignment of substitutional N-doped heterojunctions (A) N_(101)_^3^, (B) N_(101)_^3^–N_(101)_^4^, and (C) N_(101)_^3^–N_(101)_^4^–N_(101)_^7^.

**Figure 10:**
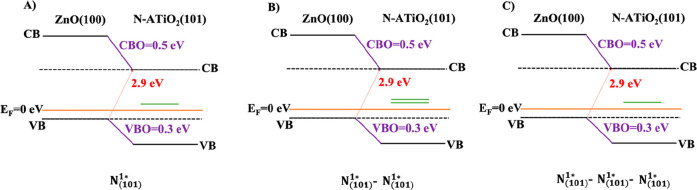
Band alignment of interstitial N-doped heterojunctions (A) N_(101)_^1_*_^, (B) N_(101)_^1_*_^–N_(101)_^1_*_^, and (C) N_(101)_^1_*_^–N_(101)_^1_*_^–N_(101)_^1_*_^.

The computed values of the VB and CB offsets of the most stable substitutional and interstitial N-doped heterojunctions above-described are reported in Figures 9 and 10, respectively. The presence of the substitutional or interstitial N dopant atom does not change the values of the VB and of the macroscopically averaged potentials (V̿) of ZnO and TiO_2_ bulks. Therefore, in the calculations of the doped ZnO(100)–ATiO_2_(101) band offsets, we have employed the E_VB_ and V̿ values of the undoped heterojunction (see Table 1).^20^ When the interface is doped with substitutional and/or interstitial N, the lineup of the macroscopically averaged electrostatic potential across the interface gives ΔV̿ = 2.3 eV, see Figure S2.

The computed values of the VB(CB) offsets of the N-doped interfaces are 0.3(0.5) eV, whereas the minimal band gaps are 2.9 eV.

**Figure 11:**
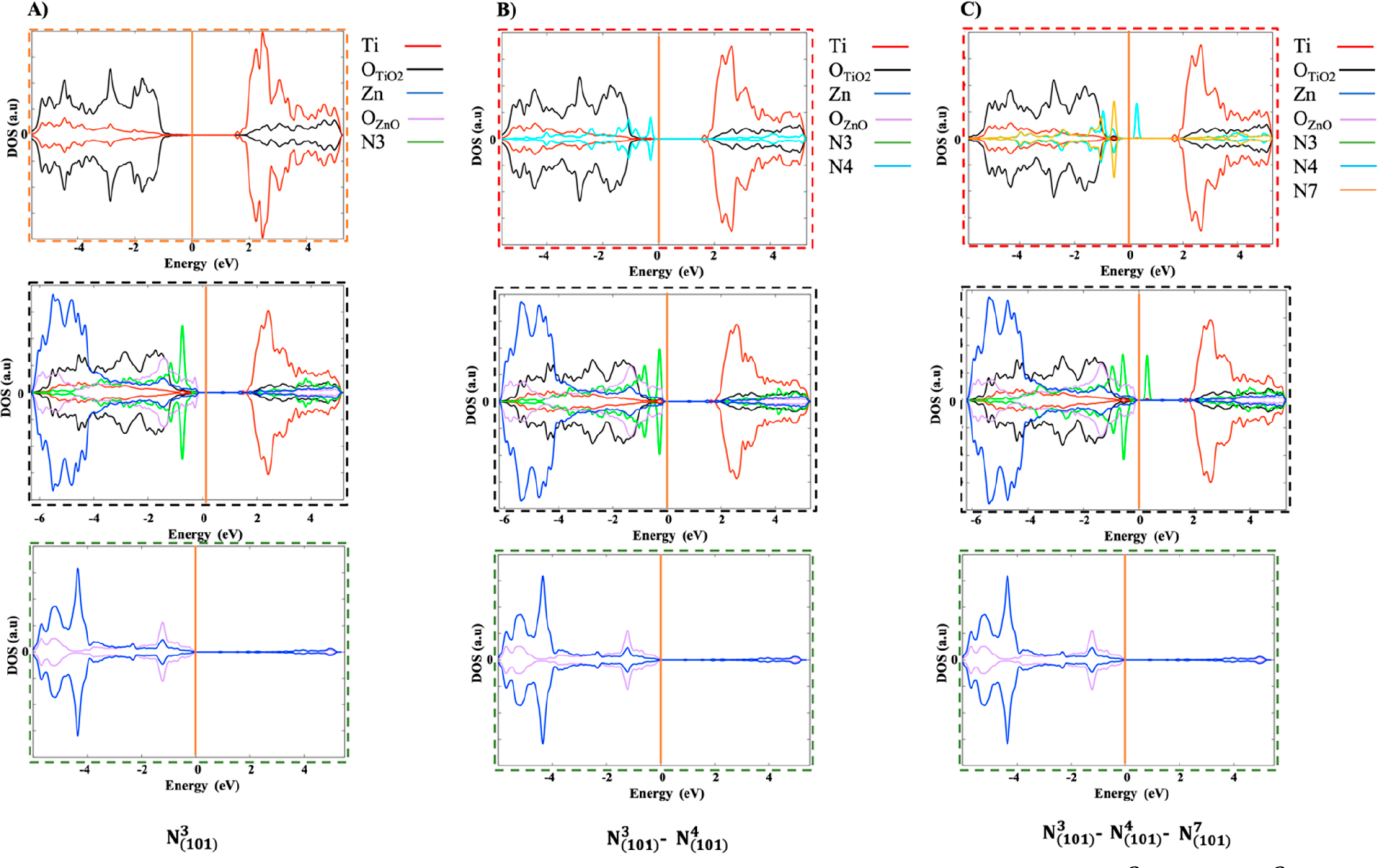
Graphical representation of substitutional N doped heterojunction (A) N_(101)_^3^, (B) N_(101)_^3^–N_(101)_^4^, and (C) N_(101)_^3^–N_(101)_^4^–N_(101)_^7^, in which ATiO_2_, ZnO(100)–ATiO_2_(101) interface, and ZnO regions outlined in orange, black, and green dashed squares, respectively, along with the PDOS. The state of titanium (Ti) and oxygen (O) in ATiO_2_ and ZnO(100)–ATiO_2_(101) interface regions are represented in red and black, respectively, while the state of zinc (Zn) and oxygen (O) in ZnO and ZnO(100)–ATiO_2_(101) interface regions are represented in blue and purple. The state of N_(101)_^3^, N_(101)_^4^, and N_(101)_^7^ in the ATiO_2_ region are represented in green, blue, and orange, respectively. On the DOS (au) axis the values above and below 0 au indicate the electrons with spin up and spin down, respectively. The Fermi energy level is represented with an orange line at 0 eV.

**Figure 12:**
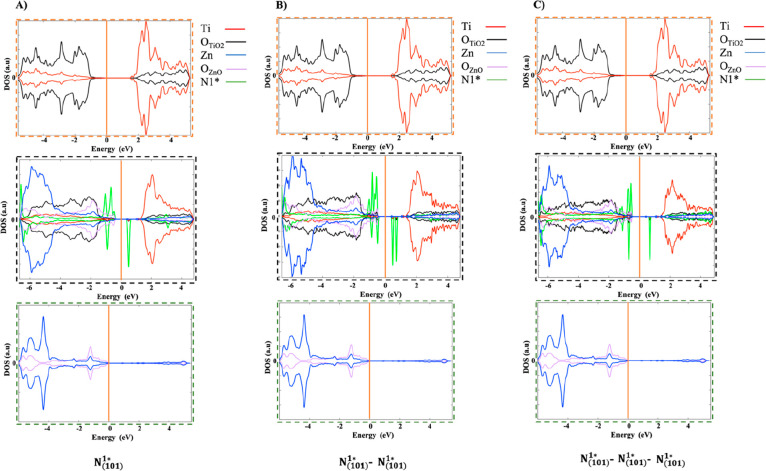
Graphical representation of interstitial N doped heterojunction (A) N_(101)_^1_*_^, (B) N_(101)_^1_*_^–N_(101)_^1_*_^, and (C) N_(101)_^1_*_^–N_(101)_^1_*_^–N_(101)_^1_*_^, in which ATiO_2_, ZnO(100)–ATiO_2_(101) interface, and ZnO regions outlined in orange, black, and green dashed squares, respectively, along with the PDOS. The state of titanium (Ti) and oxygen (O) in ATiO_2_ and ZnO(100)–ATiO_2_(101) interface regions are represented in red and black, respectively, while the state of zinc (Zn) and oxygen (O) in ZnO and ZnO(100)–ATiO_2_(101) interface regions are represented in blue and purple. The state of N_(101)_^1_*_^ in ATiO_2_ region is represented in green. On the DOS (au) axis the values above and below 0 au indicate the electrons with spin up and spin down, respectively. The Fermi energy level is represented with an orange line at 0 eV.

Figures 9 and 10 clearly indicate that the concentration and the position (substitutional and/or interstitial) of the N dopant does not affect the band offsets calculation compared to the undoped heterojunction (see Figure 3B).^20^ Anyway, in the N-doped models, substitutional and interstitial N atoms induce electronic states in the band gap due to the excess of electrons generated by the presence of the nitrogen species (see green lines in Figures 9 and 10 and PDOS in Figures 11 and 12).

In substitutional N-doping, at low N concentrations (N_(101)_^3^ and N_(101)_^3^–N_(101)_^4^ models), the generated states lie slightly above the valence band (Figures 9A,B and 11A,B). Being below the Fermi level, they correspond to partially filled orbitals occupied by the valence electrons of the N dopants. At high N concentrations (N_(101)_^3^–N_(101)_^4^–N_(101)_^7^ model), in addition to the states close to VB, there is one state higher energy above the Fermi level, indicating empty orbital (Figures 9C and 11C). Surprisingly, in the case of interstitial doping these band gap states above Fermi level are present both at low and high N concentrations (Figures 10 and 12), suggesting that the concentration of the N dopant plays a fundamental role in substitutional rather than interstitial N doping. Our results are in agreement with previous theoretical results.^5^

In all N-doped systems, the valence and conduction bands of the three regions have the same orbital character previously described for the undoped heterojunction.

**Figure 13:**
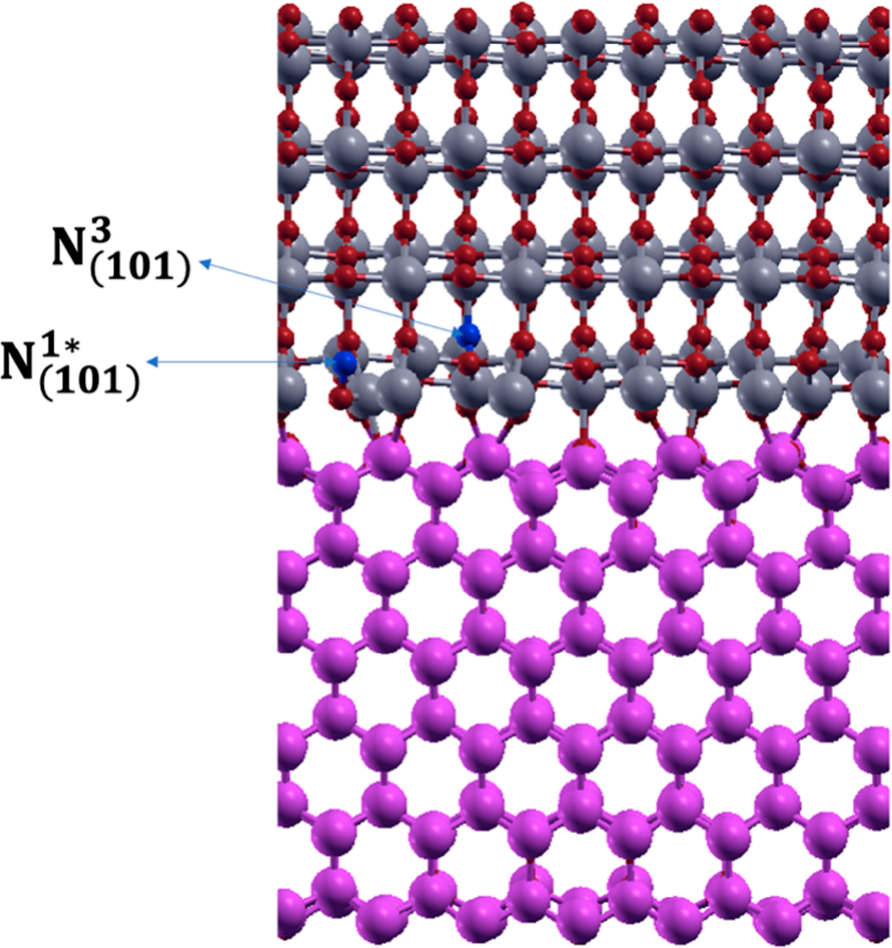
Mixed N-doped heterojunction with one substitutional N-doped site N_(101)_^3^ and one interstitial N-doped site N_(101)_^1_*_^.

With aim to simulate a real model, we have also considered a mixed N-doped heterojunction (Figure 13) focusing on the most stable substitutional and interstitial N_(101)_^3^ and N_(101)_^1_*_^ sites (see Tables in Figures 5 and 7).

**Figure 14:**
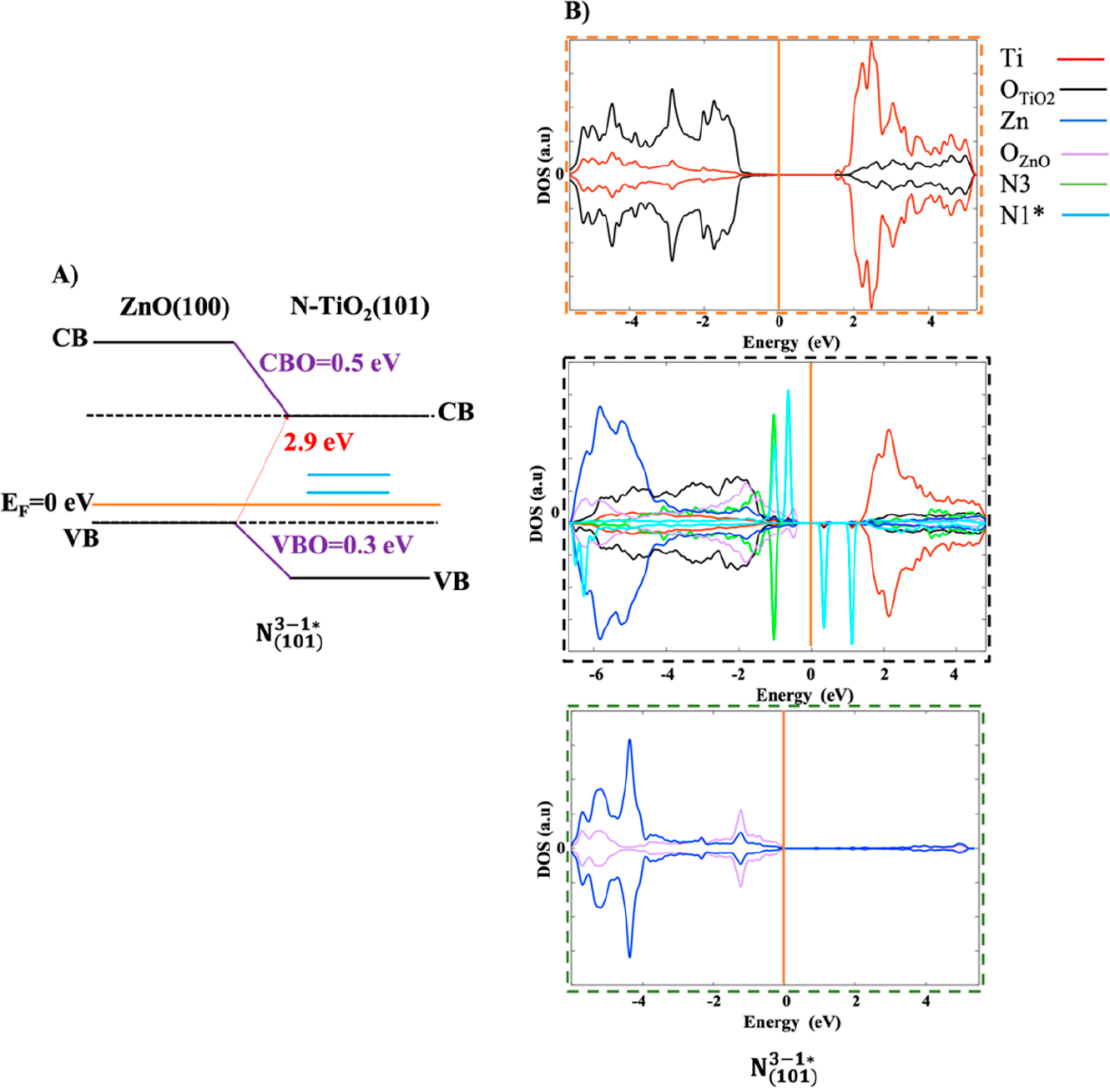
(A) Band alignment and (B) graphical representation of the mixed N-doped heterojunction N_(101)_^3–1_*_^, in which ATiO_2_, ZnO(100)–ATiO_2_(101) interface, and ZnO regions outlined in orange, black, and green dashed squares, respectively, along with the PDOS. The state of titanium (Ti) and oxygen (O) in ATiO_2_ and ZnO(100)–ATiO_2_(101) interface regions are represented in red and black, respectively, while the state of zinc (Zn) and oxygen (O) in ZnO and ZnO(100)–ATiO_2_(101) interface regions are represented in blue and purple. The state of N_(101)_^3^ and N_(101)_^1_*_^ in ATiO_2_ region are represented in green and blue, respectively. On the DOS (au) axis the values above and below 0 au indicate the electrons with spin up and spin down, respectively. The Fermi energy level is represented with an orange line at 0 eV.

The computed values of VB and CB offsets of the mixed N-doped heterojunction (N_(101)_^3–1_*_^) are reported in Figure 14A. Since the mixed N doping does not change the values of the VB and of the macroscopically averaged potentials (V̿) of ZnO and TiO_2_ bulks, for the band offsets calculations we have employed the *E*_VB_ and V̿ values of the undoped heterojunction (see Table 1). Furthermore, in the mixed N-doped heterojunction the ΔV̿ is 2.3 eV (see Figure S3) as for the N-doped models above-described (Figure S2). The computed values of the VB(CB) offsets of the mixed N-doped interface are 0.3(0.5) eV, whereas the minimal band gaps are 2.9 eV, in line with the previous results.

The PDOS plots, reported in Figure 14B, indicate the presence of two distinct partially filled states lying slightly above the valence band and generated by substitutional (green peak in Figure 14B) and interstitial N dopants (blue peak in Figure 14B), respectively. In addition, interstitial N doping induces the formation of two more higher energy empty states (see blue peaks in Figure 13A,B).

### Oxygen Vacancy (O_V_) in Undoped and N-Doped ZnO(100)–ATiO_2_(101) Heterojunction

The N-doping induces oxygen vacancies (O_V_).^64−68^ It is known that the presence of compensating defects, as oxygen vacancies, do not improve the photocatalytic performance of the N-doped ZnO.^69^ Therefore, we focus our study on the effects resulting from simultaneous presence of N impurity and O vacancy only in the ATiO_2_ portion.

Generally, the presence of N dopants in the ATiO_2_ lowers the formation energy of the oxygen vacancies (O_V_)^20,64−68,70^ because the two electrons left from the O_V_, which by locating on the Ti near to the vacant site reduce them to Ti^3+^ (donor),^1,34^ are transferred on the partially filled 2p orbitals of the N atoms (acceptor) lying close to the VB. The electron transfer from donor to acceptor atoms is favored in the presence of substitutional N-doped sites because the N 2p acceptor orbitals close to the VB hybridize with the O 2p donor orbitals. In addition the electrons flow from Ti^3+^ (donor) to N· (acceptor), forming Ti^4+^ and N^–^, stabilizes the N-doped heterojunction in the most stable singlet state.^67^

However, the position of the oxygen vacancies can affect the electronic transfer and the values of the VB and CB offsets. In fact, O_V_ far from the N-doped site generates a dipole which could affect the position of CBO and VBO.

To confirm this hypothesis, we simulated O_V_ in the (i) undoped heterojunction, (ii) heterojunction with one and two substitutional (N_(101)_^3^ and N_(101)_^3^–N_(101)_^4^) and interstitial (N_(101)_^1_*_^ and N_(101)_^1_*_^–N_(101)_^1_*_^) N dopants and (iii) the mixed N-doped models (N_(101)_^3^–N_(101)_^1_*_^).

For each system, we considered one oxygen vacancy close and far from the most stable N-doped sites, indicated for clarity O_VCLOSE_ (O_VC_) and O_VFAR_ (O_VF_), respectively. The formation energies (*E*_FORM_, eV), computed by using eq 5, are reported in Table 2.

**Table 2:** Formation Energies (*E*_FORM_, eV) of the Oxygen Vacancy (O_V_) Close (O_VC_) and Far (O_VF_) from the Substitutional and Interstitial N-Doped Sites Compared with the *E*_FORM_ of the O_V_ in the Undoped System

*E*_FORM_ (eV)	O_VC_	O_VF_
ZnO(100)–ATiO_2_(101)	2.16	2.46
*N*_(101)_^3^	1.79	2.32
*N*_(101)_^1_*_^	2.51	3.62
*N*_(101)_^3^–*N*_(101)_^4^	1.05	1.71
N_(101)_^1_*_^–*N*_(101)_^1_*_^	1.80	2.52
*N*_(101)_^3^–*N*_(101)_^1_*_^	1.45	2.64

**Figure 15:**
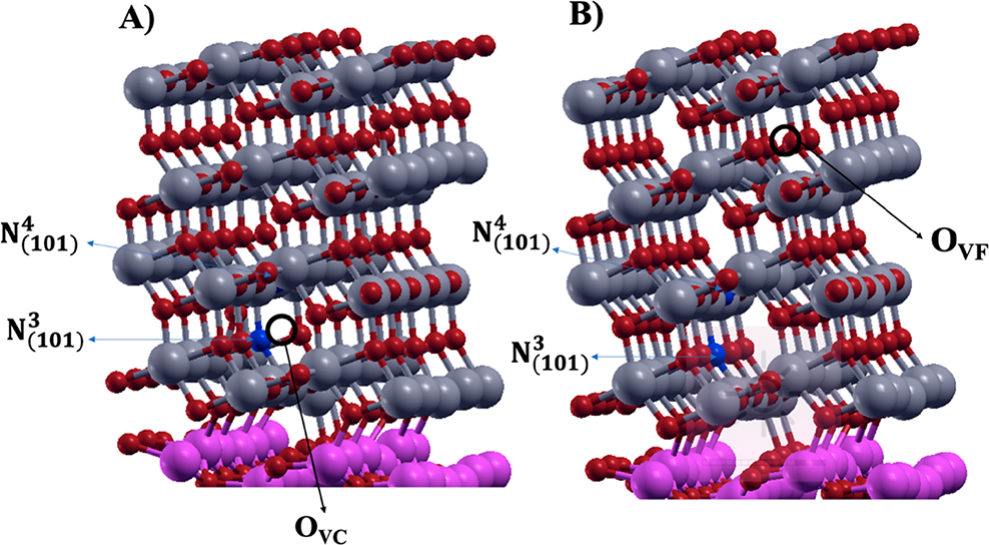
N_(101)_^3^–N_(101)_^4^ heterojunction with (A) O_V_ close and (B) O_V_ far to the substitutional N-doped sites.

The data reported in Table 2 indicate that (i) the formation energy of O_VC_ is lower than that of O_VF_ and (ii) O_VC_ and O_VF_ are formed more easily in the presence of substitutional N-doped sites due to the greater hybridization of the N 2p and O 2p orbitals compared to the interstitial N-doping (see Figures 11 and 12). In particular, the *E*_FORM_ of both vacancies is reduced in the substitutional N-doped heterojunction N_(101)_^3^–N_(101)_^4^ (see Table 2). In fact, the electron transfer from the two Ti^3+^ to N_(101)_^3^ and N_(101)_^4^ stabilizes the N-doped heterojunction in a singlet state with energy lower than a triplet state. For this reason, all the analysis will be performed on these two systems, which optimized structures are reported in Figure 15.

**Figure 16:**
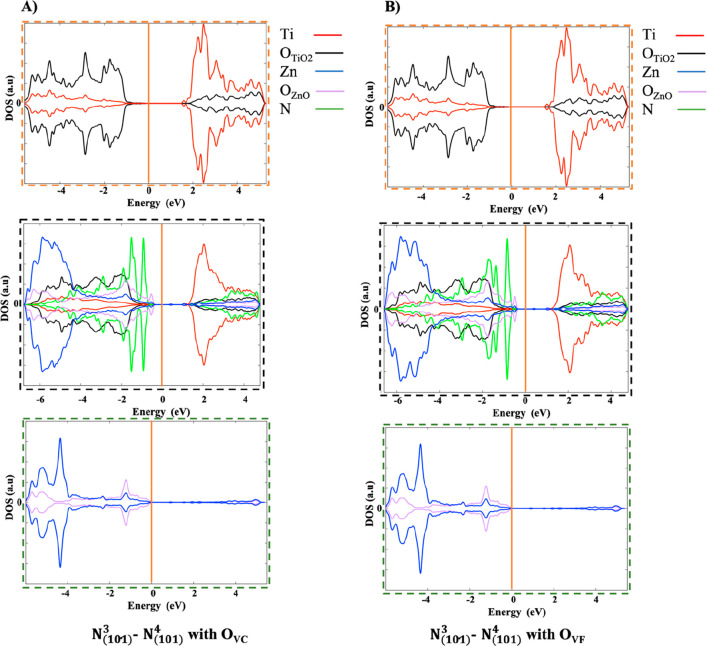
Graphical representation of the N_(101)_^3^–N_(101)_^4^ heterojunction with one oxygen vacancy (O_V_) (A) close (O_VC_) and (B) far (O_VF_) from the substitutional N-doped site, in which ATiO_2_, ZnO(100)–ATiO_2_(101) interface, and ZnO regions outlined in orange, black, and green dashed squares, respectively, along with the PDOS. The state of titanium (Ti) and oxygen (O) in ATiO_2_ and ZnO(100)–ATiO_2_(101) interface regions are represented in red and black, respectively, while the state of zinc (Zn) and oxygen (O) in ZnO and ZnO(100)–ATiO_2_(101) interface regions are represented in blue and purple. The state of N_(101)_^3^ and N_(101)_^4^ in ATiO_2_ region are represented in green. On the DOS (au) axis the values above and below 0 au indicate the electrons with spin up and spin down, respectively. The Fermi energy level is represented with an orange line at 0 eV.

The PDOS plots of O_VC_ and O_VF_ in the presence of two substitutional N-doping sites, reported in Figure 16A and B, respectively, indicate the presence of states lying slightly above the valence band and below the Fermi level (see green peaks in Figure 16). Contrary to the cases above-described, these peaks correspond to completely full orbitals occupied by the electrons of the N dopants and those left by the vacancies. In fact, the electrons generated by the oxygen vacancy spontaneously migrate toward the states generated by the dopants, forming two N^–^ diamagnetic centers and a heterojunction in which there are no unpaired electrons, as confirmed by the spin density plots.

**Figure 17:**
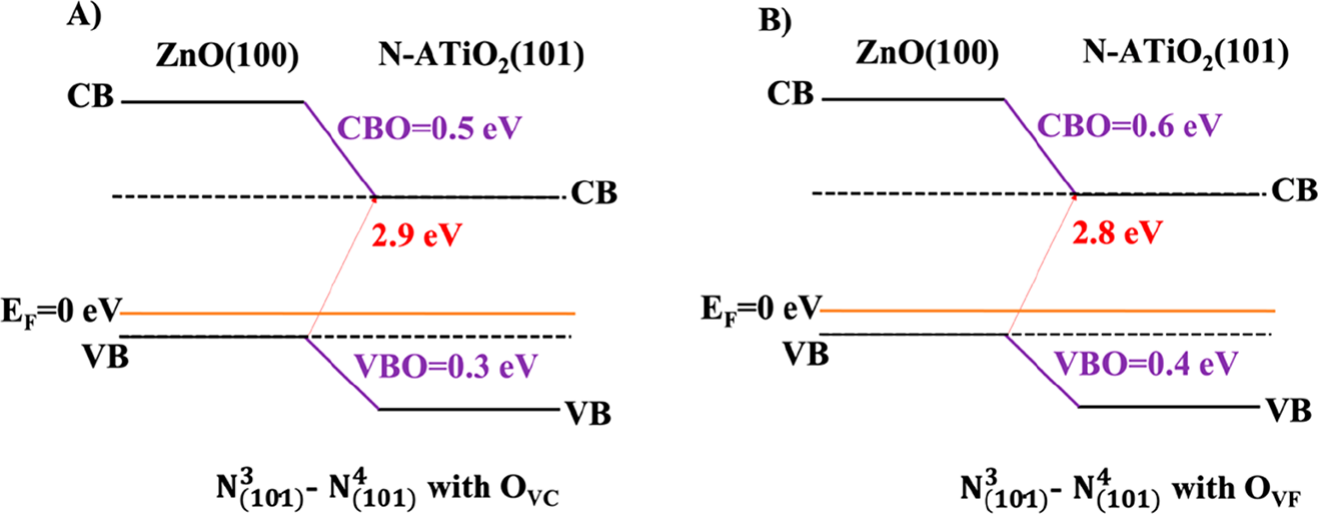
Band alignment of the N_(101)_^3^–N_(101)_^4^ heterojunction with one oxygen vacancy (O_V_) (A) close (O_VC_) and (B) far (O_VF_) from the substitutional N-doped sites.

After this electron transfer, the VB and CB offsets computed for N_(101)_^3^–N_(101)_^4^ heterojunction with O_VC_(O_VF_) are equal to VBO = 0.3(0.4) eV and CBO = 0.5(0.6) eV (Figure 17A,B). In both cases, VBO and CBO have been defined using the *E*_VB_ and V̿ values reported in Table 1 and a ΔV̿ equal to 2.3 eV (see Figure S4).

For O_VC_ and O_VF_ the values of VBO and CBO are very similar to those computed for the undoped and N-doped heterojunctions, respectively (see Figures 3A, 9, and 10), suggesting that the presence and position of the oxygen vacancy does not affect the band alignment and the electronic characteristics of the system. However, the combination of N impurity and the O vacancy (i) improves the electron transfer to DONOR to ACCEPTOR atoms, (ii) stabilizes the heterojunctions in a singlet state more stable than a triplet one, and (iii) increases the absorption response in the visible region.

## Conclusion

In this work, we have investigated the electronic and structural properties of the undoped and N-doped ZnO(100)–ATiO_2_(101) heterojunction by means of density functional theory (DFT) with the aim of understand how the combined system, the N-doping and the presence of oxygen vacancies (O_V_) can improve the photocatalytic activity of the two separate oxide semiconductors. Our undoped model predicts the formation of two interfaces, defined right and left interface, with the right interface energetically more stable than the left one and as a consequence selected for our theoretical analysis. The analysis of the electronic properties (band alignment, PDOS and interface dipole) confirm that the undoped ZnO(100)–ATiO_2_(101) is a type-II heterojunction, in which electrons and holes, generated by photocatalysis process, migrate from the CB of the ZnO to the CB of the ATiO_2_ and from the VB of ATiO_2_ to the VB of ZnO, respectively. Therefore, heterojunction turns out as crucial to prevent electron–hole recombination and consequently to improve the photocatalytic performance and electronic properties of semiconductors.

Moreover, calculations suggested that, to further enhance the photocatalytic activity of the ZnO(100)–ATiO_2_(101) heterojunction, the formation of N-doped sites should be considered. In detail, the formation of substitutional N dopants is favored in the ATiO_2_ portion with the formation energy value decreases moving from the interface to the inside of the anatase region as a consequence of the destabilizing structural rearrangements. Vice versa, the formation of interstitial N-doped sites is energetically favored in the ZnO region due to stabilizing structural rearrangements in wurtzite portion. In addition, the energy of the doped interface increases when the interstitial N atom is placed farther from the interfacial region. Starting from the most stable configuration with substitutional and interstitial N-doped sites, we have investigated how the N-dopants concentration can influence the electronic structure of these heterojunctions. In all considered models, the presence of N-doped sites generates band gap states both at low and high N concentration. At low N concentration, these states are slightly above the valence band (VB) and, being the dopant a nitrogen atom with five valence electrons, the 2p orbitals are partially filled. At high N concentrations, in addition to the states close to the VB, there are some empty states higher in the gap, which confirm the greater photocatalytic activity of the N-doped heterojunctions compared to undoped ones under visible light. In fact, these empty states act as a deep electronic trap, generating a better charge separation and delaying electron–hole recombination. It is interesting to note that in the case of interstitial N-doping, the systems are already active at low N concentration. This suggests that the concentration of the N dopant plays a fundamental role in substitutional rather than interstitial N doping. Furthermore, the N dopants presence facilitates the formation of oxygen vacancies, especially if O_V_ are close (O_VC_) to the N-doped sites. In fact, the electrons left by the oxygen vacancy spontaneously migrate toward the partially filled states of the N atoms, leading a decrease in the formation energy (*E*_FORM_) of the vacancy. Finally, we have shown that the band alignment of the N-doped models, compared to the undoped system, is not affect neither by the N presence nor by the N concentration or by the presence of vacancies close and/or far from the N-dopant sites. Our results provide key and novel information about the photocatalytic activity of the ZnO(100)–ATiO_2_(101) heterojunction, which improves in the presence of N-doping and oxygen vacancies. Our study paves the way for a new and successful synthetic strategy to obtain TiO_2_-based semiconductors with enhanced catalytic activities in the visible light. Therefore, based on our theoretical results, experimental activities concerning the synthesis and photocatalytic applications of undoped and N-doped heterojunctions are in progress.

## Supporting Information Available

Projected density of state (PDOS) and planar-averaged electrostatic potential and its macroscopic average for TiO_2_ bulk and ZnO bulk. Planar-averaged electrostatic potential and its macroscopic average for (i) more stable substitutional and interstitial N-doped sites, (ii) mixed, and (iii) N_(101)_^3^–N_(101)_^4^ heterojunction with oxygen vacancies heterojunction (PDF)
