# Supplementary material for: DFT Investigation of Substitutional and Interstitial Nitrogen-Doping Effects on a ZnO(100)–TiO2(101) Heterojunction
Source: J Phys Chem C Nanomater Interfaces. 2022 Feb 3;126(6):3180–93. doi: 10.1021/acs.jpcc.1c09395 (PMC9946291; doi:10.1021/acs.jpcc.1c09395)
Supplement: Supplementary file 1 — jp1c09395_si_001.pdf [file jp1c09395_si_001.pdf]

Supporting Information

A DFT Investigation of Substitutional and Interstitial Nitrogen (N)-Doping Effects on a ZnO(100)-TiO_2(101) Heterojunction.

Ida Ritacco[a], Olga Sacco[a], Lucia Caporaso[a], Matteo Farnesi Camellone[b]*

[a] Dipartimento di Chimica e Biologia, Università degli Studi di Salerno, via Giovanni Paolo II 132, 84084 Fisciano, Salerno, Italy

[b] CNR-IOM, Consiglio Nazionale delle Ricerche - Istituto Officina dei Materiali, c/o SISSA, 34136 Trieste, Italy

E-mail: lcaporaso@unisa.it

Table of Content

Figure S1. Projected Density of State (PDOS), Planar-averaged electrostatic potential and its macroscopic average for TiO_2 bulk and ZnO bulk.

Pag.S2

Figure S2. Planar-averaged electrostatic potential and its macroscopic average for $N^{3}_{(101)}$(A) and $N^{1*}_{(101)}$ heterojunctions.

Pag.S3

Figure S3. Planar-averaged electrostatic potential and its macroscopic average for ($N^{3-1*}_{(101)}$) heterojunction.

Pag.S4

Figure S4. Planar-averaged electrostatic potential and its macroscopic average for $N^{3}_{(101)}$-$N^{4}_{(101)}$ heterojunction with oxygen vacancies.

Pag.S5

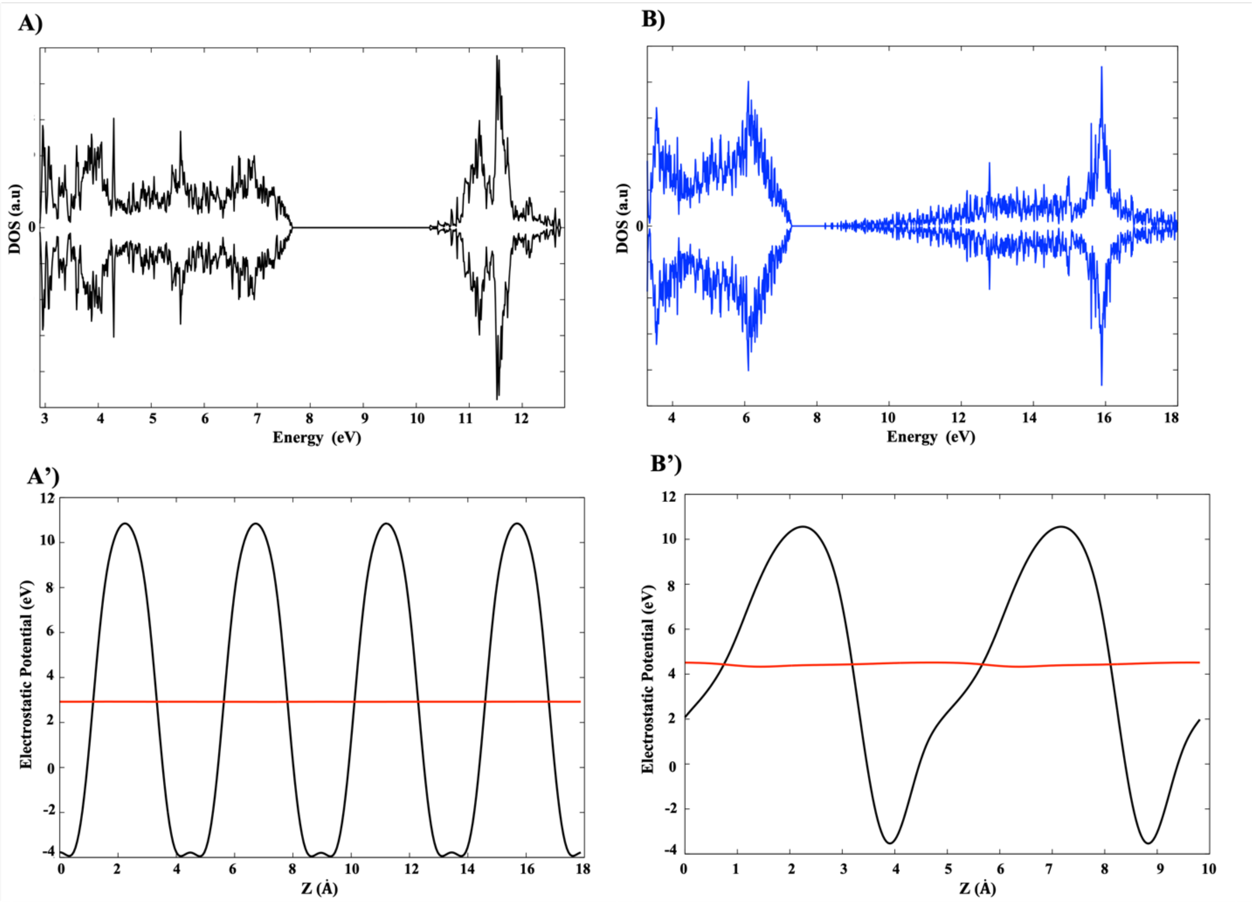

Figure S1. Projected Density of State (PDOS), Planar-averaged electrostatic potential (black line) and its macroscopic average (red line) for TiO_2 bulk (A and A', respectively) and ZnO bulk (B and B', respectively).

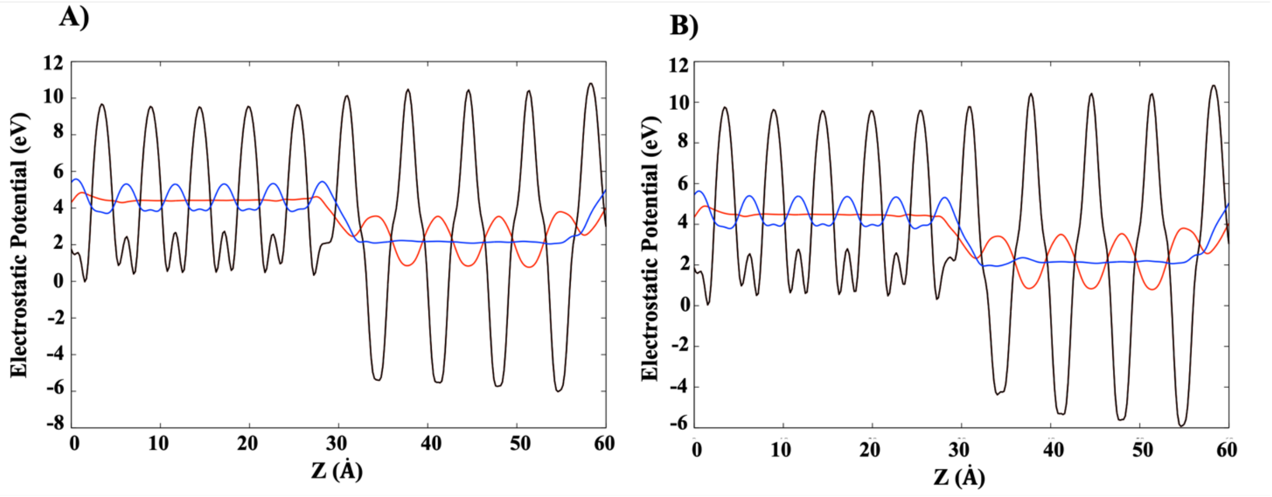

Figure S2. Planar-averaged electrostatic potential (black line) and its macroscopic average (red and blue lines, respectively) for $N^{3}_{(101)}$(A) and $N^{1*}_{(101)}$(B) heterojunctions.

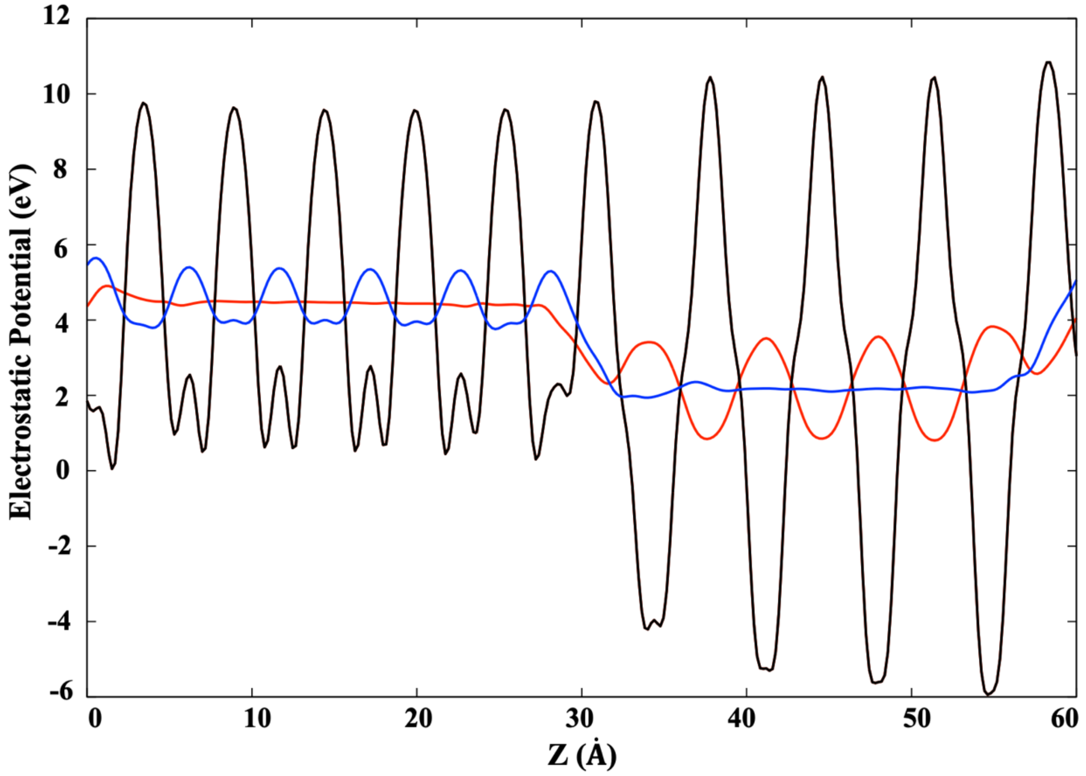

Figure S3. Planar-averaged electrostatic potential (black line) and its macroscopic average for ($N^{3-1*}_{(101)}$) heterojunction (red and blue lines, respectively).

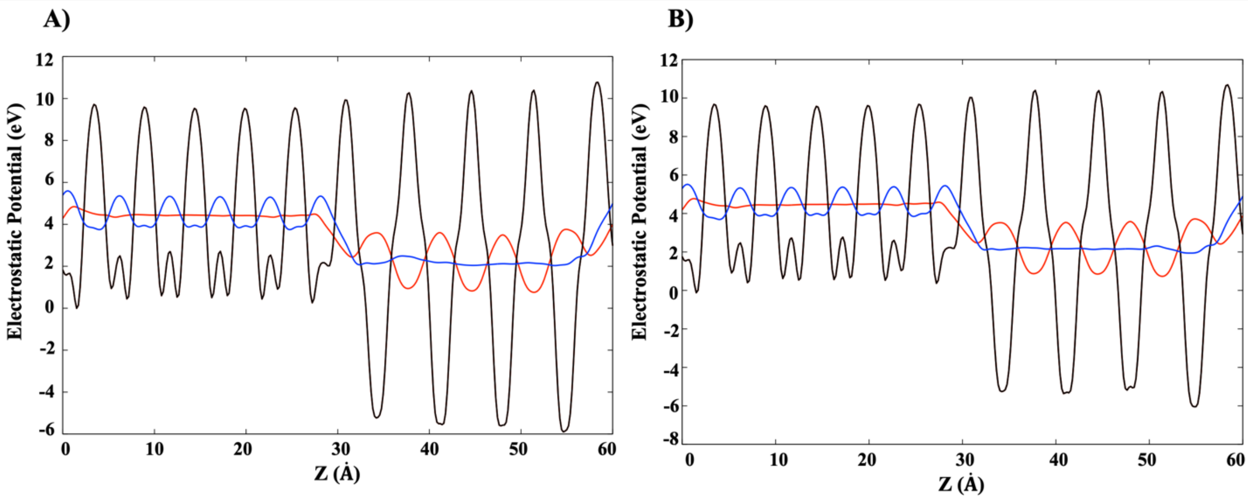

Figure S4. Planar-averaged electrostatic potential (black line) and its macroscopic average (red and blue lines, respectively) for $N^3_{(101)}$-$N^4_{(101)}$ heterojunction with A) O_V close and B) O_V far to the substitutional N-doped sites.
